# Long-Term Zinc Supplementation Improves Liver Function and Decreases the Risk of Developing Hepatocellular Carcinoma

**DOI:** 10.3390/nu10121955

**Published:** 2018-12-10

**Authors:** Atsushi Hosui, Eiji Kimura, Sumiko Abe, Takashi Tanimoto, Kousaku Onishi, Yukihiro Kusumoto, Yuka Sueyoshi, Kengo Matsumoto, Motohiro Hirao, Takuya Yamada, Naoki Hiramatsu

**Affiliations:** Department of Gastroenterology and Hepatology, Osaka-Rosai Hospital, 1179-3 Nagasone, Kitaku, Sakai, Osaka 591-8025, Japan; eijikimura0516@gmail.com (E.K.); Abe@osakah.johas.go.jp (S.A.); I-will-allow-xxx@softbank.ne.jp (T.T.); k-ohnishi@osakah.johas.go.jp (K.O.); y.kusumoto@osakah.johas.go.jp (Y.K.); sueyoshi@osakah.johas.go.jp (Y.S.); k-matsumoto@osakah.johas.go.jp (K.M.); motty@osakah.johas.go.jp (M.H.); yamada@osakah.johas.go.jp (T.Y.); hiramatsu@osakah.johas.go.jp (N.H.)

**Keywords:** zinc, HCC, liver function, Zn concentration

## Abstract

Zinc plays a pivotal role in various zinc enzymes, which are crucial in the maintenance of liver function. Patients with chronic liver diseases (CLDs) usually have lower concentrations of zinc, which decrease further as liver fibrosis progresses. Whether long-term zinc supplementation improves liver function and reduces the risk of hepatocellular carcinoma (HCC) development remains unknown. Two hundred and sixty-seven patients with CLDs who received a zinc preparation (Zn-group; 196 patients), or who did not receive zinc (no Zn-treatment group; 71 patients), were retrospectively analyzed in this study. The Zn-group was divided into 4 groups according to their serum Zn concentrations at 6 months after the start of Zn treatment. Liver function significantly deteriorated in the no Zn-treatment group, while no notable change was observed in the Zn-group. The cumulative incidence rates of events and HCC at 3 years were observed to be lower in the Zn-group (9.5%, 7.6%) than in the no Zn-treatment group (24.9%, 19.2%) (*p* < 0.001). According to serum Zn concentrations, the cumulative incidence rates of events and HCC were significantly decreased in patients with Zn concentrations ≥ 70 µg/dL (*p* < 0.001). Zinc supplementation appears to be effective at maintaining liver function and suppressing events and HCC development, especially among patients whose Zn concentration is greater than 70 µg/dL.

## 1. Introduction

Zinc, an essential trace element, has been reported to play various pivotal roles in the human body. Notably, in the liver, zinc is needed for the activation of many enzymes, such as ornithine transcarbamylase (OTC) and glutamate dehydrogenase (GDH), which are utilized in the urea cycle and the glutamine synthetase cycle respectively. Superoxide dismutase (SOD), which requires zinc for its activation, has strong antioxidant activity. Zinc deficiency may cause SOD inactivity, followed by increased reactive oxygen species (ROS). Takeda et al. recently reported that zinc deficiency delays both extracellular adenosine triphosphate (ATP) clearance and adenosine generation, followed by enhanced inflammation [1]. Many roles of zinc, especially in chronic liver diseases (CLDs), were explained by Himoto in a review article [2].

Vallee et al. first reported on the occurrence of marked hypozincemia in patients with severe cirrhosis in 1956 [3]. This finding has been confirmed by many investigators [4,5]. The mechanisms underlying zinc deficiency in patients with CLDs appear to be multifactorial. First, patients with CLDs have inadequate dietary intake of zinc [6]. Second, the absorption of zinc is also impaired in these patients because of portal hypertension [7]. Third, urinary excretion of zinc is increased, and greater excretion has been observed in patients taking diuretics [4,5,8,9]. Based on these results, the serum zinc concentration decreases with the progression of CLDs.

Limited clinical trials have evaluated the effects of zinc supplementation on clinical outcomes in patients with CLDs. As zinc helps activate some enzymes in the urea cycle, many investigators have focused on improving hepatic encephalopathy by administering a zinc supplement. Reding et al. first reported that oral zinc supplementation improved hepatic encephalopathy in 22 patients with cirrhosis in a double-blind randomized trial. The effect of taking zinc acetate, 600 mg a day for 7 days, was evaluated in that study [10]. Katayama et al. also presented the importance of zinc supplementation in a trial showing that treatment with zinc acetate for 3 months was an effective and safe treatment for hyperammonemia in patients with liver cirrhosis [11]. A systematic review and meta-analysis of the use of zinc in patients with hepatic encephalopathy revealed a significant improvement in psychological tests after zinc therapy [12]. However, many of these studies have focused on the short-term administration of zinc. The period of zinc administration was only 7 days in the study by Reding and colleagues, and 3 months in the study by Katayama and coworkers. Few papers have explored the long-term effects of zinc on patients with CLDs. Therefore, the first issue is whether long-term zinc administration improves the long-term prognosis of these patients. The second issue is determining the zinc concentration necessary to improve the prognosis of patients with CLDs [10,11,12]. While all of these reports supported the efficacy of zinc supplementation in patients with CLDs, almost all studies explored the efficacy of zinc by comparing the placebo group with the zinc group; thus, the appropriate plasma zinc concentration after zinc administration to achieve these effects remains unknown.

In the present study, we investigated liver function and the risk of events—death, liver failure and the development of hepatocellular carcinoma (HCC)—after the administration of zinc for more than 3 years, to clarify the long-term effects of zinc. In addition, the incidence rates of events were evaluated among the groups stratified according to their zinc concentrations measured after 6 months of zinc administration. Zinc preparations were approved in 13 February 2018, and they may become valuable drugs for the prevention of cancer.

## 2. Patients and Methods

267 patients with CLDs, who had received a zinc preparation (ZnSO4, Zn 60–120 mg/day; Zn-group; 196 patients) for at least 6 months or who had never received zinc (no Zn-treatment group; 71 patients), were retrospectively analyzed in this study. These patients were initially diagnosed with CLDs at Osaka Rosai Hospital between 1 January 2007 and 31 December 2017. A total of 267 consecutive patients who provided informed consent were recruited in this study. Each doctor decided whether to administer zinc supplements, but some physicians tended to administer them to patients with severe fibrosis. The dose of zinc was determined according to the decision of the primary physician, and did not depend on the clinical situation or disease severity. Patients in the Zn-group were divided into 4 groups according to their Zn concentration at 6 months after the start of Zn treatment—less than 50 µg/dL (G1), 50–69 µg/dL (G2), 70–89 µg/dL (G3), and not less than 90 µg/dL (G4). Liver function and the number of events (death, development of liver cancer, and appearance of liver failure) were evaluated at least every 6 months. The severity of liver disease was assessed based on the Child-Pugh score, and patients were grouped into Class A, B and C according to a total score of 5–6, 7–9 or 10–15. The total bilirubin (T-Bil), albumin (Alb), and prothrombin (PT) values, and the presence/absence of ascites and hepatic encephalopathy, were scored as 1, 2, or 3. A fasting blood sample (3–4 mL) was drawn from each subject in plain, EDTA, and PT vials to perform the following investigations: Aspartate aminotransferase (AST), alanine aminotransferase (ALT), T-Bil, Alb, and branched amino acids/tyrosine ratio (BTR), which were performed on a fully automated analyzer (Olympus AU 400, Olympus Corporation, Tokyo, Japan). The plasma concentration of zinc was assessed on an atomic absorption spectrophotometer (Shino-test Company, Tokyo, Japan). This study was approved by the Ethics and Committee of Osaka-Rosai Hospital (approval code 29–47). All participants provided written informed consent.

The data are presented as the means ± standard error (SE). Data from two groups were compared using unpaired *t*-tests. Multiple comparisons were performed by ANOVAs with the Scheffe post hoc test. A value of *p* < 0.05 was considered statistically significant. The log-rank test was used to assess the cumulative incidence rates of events and HCC development.

## 3. Results

### 3.1. Zinc Administration Improved Liver Function

**Figure 1:**
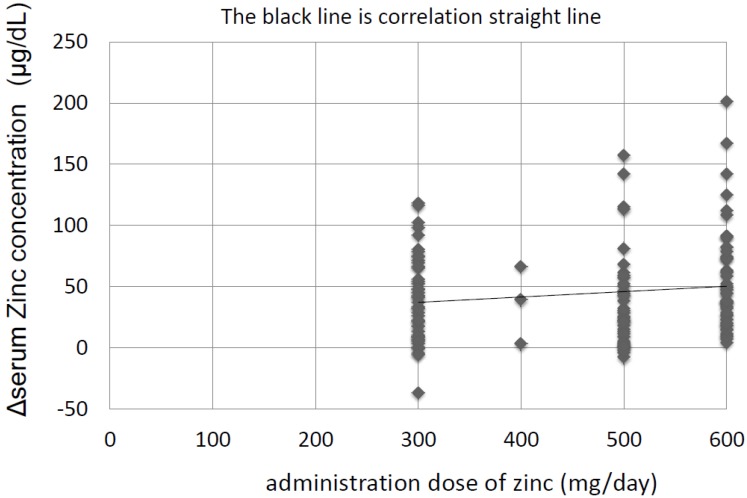
Relationships between the administered dose of zinc and the increase in serum zinc concentration. ΔZn = Zn concentration (6 months after treatment) − Zn concentration (before treatment), ΔZn = 0.044 × (Zn dose) + 23.8, *R* = 0.14, *p* = 0.023. A weak relationship exists between the administered dose of zinc and the increase in serum zinc concentration.

**Figure 2:**
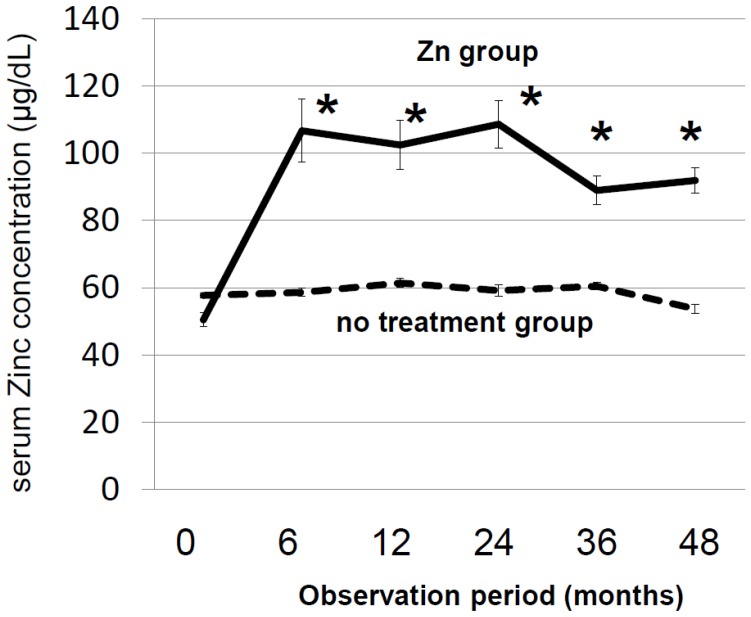
Serial changes in the serum zinc concentration in the Zn- and no Zn-treatment groups. Asterisks indicate significant differences (*, *p* < 0.001 compared to the value recorded before treatment). The zinc concentration increased to greater than 90 µg/dL in the Zn-group, and was maintained at that level for 4 years.

**Figure 3:**
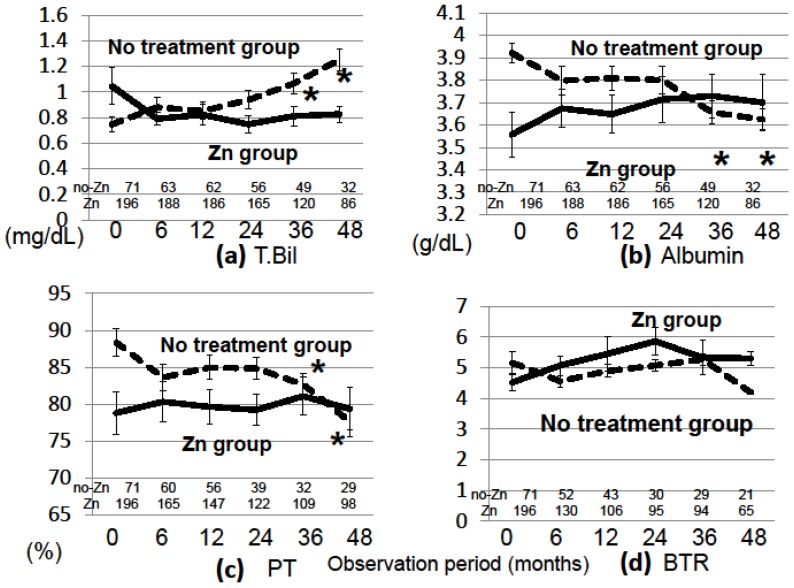
Serial changes in laboratory variables in the Zn- and no Zn-treatment groups. (**a**) Total bilirubin, (**b**) albumin, (**c**) % prothrombin time, (**d**) branched chain amino acids/tyrosine ratio, Asterisks indicate significant differences (*, *p* < 0.01, compared to the value measured before treatment in each group). The number of patients who underwent blood tests is shown at the bottom of each graph.

The characteristics of the 267 patients with CLDs are shown in Table 1. The patients in the Zn-group were older and had more advanced liver diseases than patients in the no Zn-treatment group; the proportion of patients with cirrhosis and the proportion of patients with Child-Pugh scores of B and C was higher in the Zn-group than in the no Zn-treatment group: cirrhosis: 52.6% vs. 21.1% (*p* < 0.001), Child-Pugh-B and C: 50.0% vs. 5.6% (*p* < 0.001). Other characteristics of the two groups are shown in Supplementary Table S1. The number of patients taking diuretics was greater in the Zn-group than in the no Zn-treatment group, but this did not seem to influence the outcomes. The mean follow-up period was 40.0 ± 31.5 months, and patients took zinc for the entire follow-up period. Regarding the relationship between the dose of zinc administered and the increase in zinc concentration, the zinc concentration increased to a greater extent when higher doses of zinc were administered (Figure 1). The zinc concentration increased to greater than 90 µg/dL in the Zn-group and was maintained at that level for 4 years, while it did not increase and remained low in the no Zn-treatment group (Figure 2). The Zn concentration remained in the plateau phase after 6 months of Zn treatment; thus, the Zn concentration at 6 months after the start of Zn therapy was used to investigate the relationship between the Zn concentration and prognosis. The biochemical changes in T-Bil levels, Alb levels, PT activity, and BTR are shown in Figure 3. Before treatment, the T-Bil level was high, and the Alb and PT activity levels were low in the Zn-group. The T-Bil level in the no Zn-treatment group significantly increased after 3 years (*p* < 0.01), while in the Zn-group, it remained at approximately the same level (Figure 3a). In the no Zn-treatment group, the levels of Alb and PT activity were significantly decreased after 3 years (*p* < 0.01), while in the Zn-group, they remained at approximately the same levels (Figure 3b,c). Taken together, liver function significantly deteriorated in the no Zn-treatment group, while no notable change was observed in the Zn-group. This suggests that zinc administration can prevent the worsening of liver function in patients with CLDs. AST and ALT levels gradually decreased after treatment with zinc preparations (Supplementary Figure S1).

### 3.2. Zinc Administration May Suppress the Incidence of HCC

**Figure 4:**
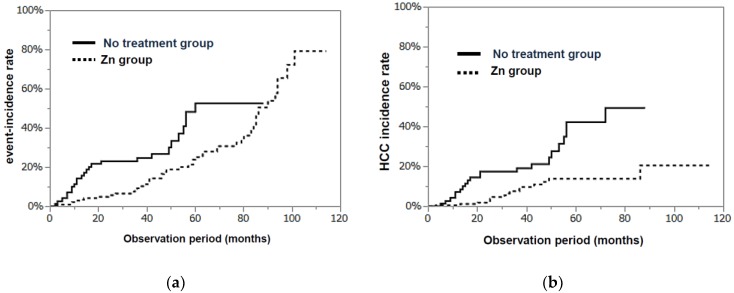
(**a**) The cumulative incidence rates of events (death, the development of HCC, and appearance of liver failure) in the Zn- and no Zn-treatment groups (log-rank test *p* = 0.0049). (**b**) The incidence rates of HCC in both groups (log-rank test, *p* = 0.0002).

As shown in Figure 4a, the cumulative incidence rate of events (death, the development of liver cancer, and appearance of liver failure) at 3 years was 9.5% in the Zn-group, which was significantly less than that in the no Zn-treatment group (24.9%, *p* = 0.0049). In particular, the incidence rate of HCC (3 years) was significantly lower in the Zn-group (7.6%) than in the no Zn-treatment group (19.2%, *p* = 0.0002; Figure 4b).

### 3.3. Patients with High Zinc Concentrations after Zn Therapy Had Lower Cumulative Event and HCC Incidence Rates

**Figure 5:**
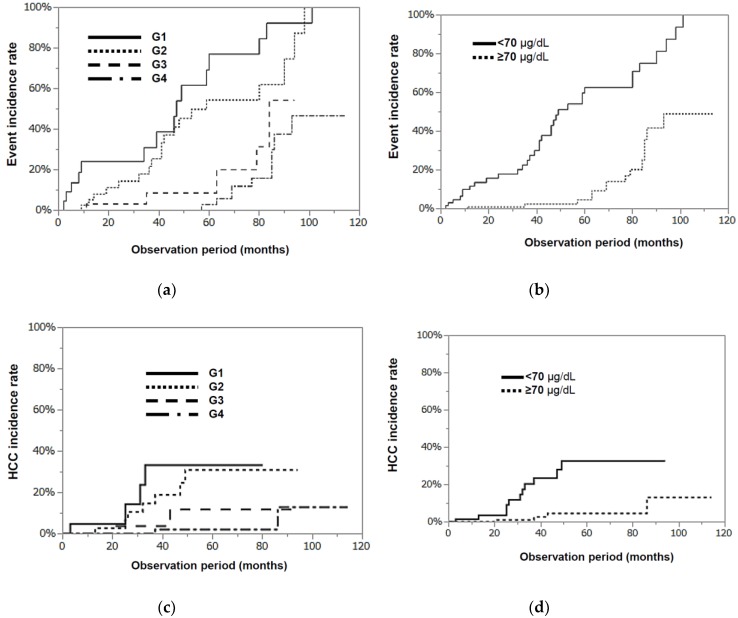
The cumulative incidence rates of events (**a**) and the incidence rates of HCC (**c**) in the 4 groups stratified according to their serum zinc concentrations measured 6 months after zinc administration—less than 50 µg/dL (G1), 50–69 µg/dL (G2), 70–89 µg/dL (G3), and not less than 90 µg/dL (G4). (**a**) G1/G2 groups vs. G3/G4 groups, *p* < 0.0001, and (**c**) G1/G2 groups vs. G3/G4 groups, (*p* = 0.0007). (**b**,**d**) The cumulative incidence rates of events (**b**) and the incidence rates of HCC (**d**) in 2 groups stratified according to their serum zinc concentrations measured 6 months after zinc administration (less than 70 µg/dL (lower zinc group) and not less than 70 µg/dL (higher zinc group)); (**b**) *p* < 0.0001 and (**d**) *p* < 0.0001).

**Figure 6:**
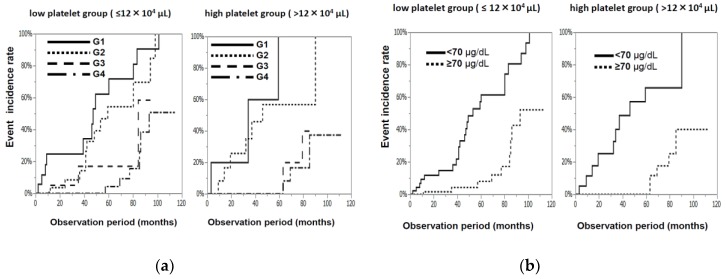

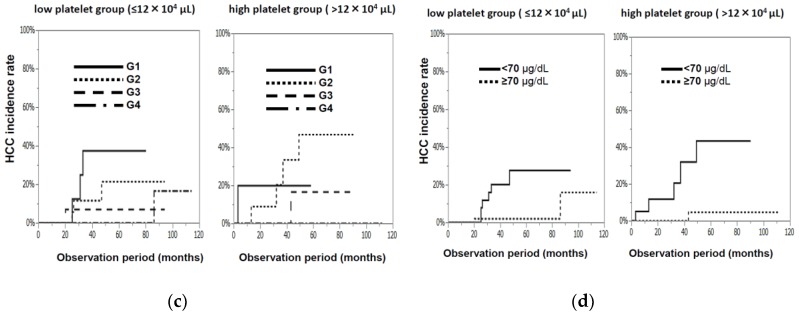
The cumulative incidence rates of events (**a**) and the incidence rates of HCC (**c**) (left panel) in the low-platelet count group and (right panel) the high-platelet count group ((**a**) in both panels, G1/G2 groups vs. G3/G4 groups, *p* < 0.0001, (**c**) left panel, G1/G2/G3 groups vs. G4 group, *p* = 0.0172, and right panel, G1/G2 groups vs. G3/G4 groups, *p* = 0.0098). (**b**,**d**) The cumulative incidence rates of events (**b**) and HCC (**d**) (left panel) in the low-platelet count group and (right panel) the high-platelet count group, (low serum zinc concentration group and high serum zinc concentration group) (all panels, *p* < 0.0001).

Oral zinc supplementation was shown to be quite effective for patients with CLDs. The Zn-group was divided into 4 groups according to their Zn concentration at 6 months after the start of Zn treatment—less than 50 µg/dL (G1), 50–69 µg/dL (G2), 70–89 µg/dL (G3), and not less than 90µg/dL (G4)—to clarify the serum zinc concentration needed to achieve a positive effect. The characteristics of these 4 groups are shown in Table 2. The G1 group tended to be older and had lower platelet counts than the other three groups, but no significant differences were observed in the 4 groups with regard to age, gender, Zn concentration, or the levels of T-Bil, Alb, and PT activity before Zn administration. The cumulative incidence rates of events recorded at 3 years were 34.5%, 21.7%, 8.6%, and 0.0% in the G1, G2, G3, and G4 groups, respectively (Figure 5a), and they were significantly lower (*p* < 0.0001, Figure 5b) in the G3 and G4 groups (not less than 70 µg/dL) than in the G1 and G2 groups (less than 70 µg/dL). Regarding the relationship between the serum zinc concentration and the incidence rate of HCC, the HCC incidence rates recorded at 3 years were 33.3%, 16.7%, 3.8%, and 0.0% in the G1, G2, G3, and G4 groups, respectively (Figure 5c), and the HCC incidence rate was significantly lower in the G3 and G4 groups than in the G1 and G2 groups (*p* < 0.001, Figure 5d). The progression of fibrosis has been reported to cause HCC, and the Zn-group was divided into 2 groups according to their platelet counts before treatment (borderline: 12 × 10^4^ platelets/µL). As shown in Figure 6a,b, regardless of whether the original platelet counts were high or low, significantly lower cumulative incidence rates (*p* < 0.0001) of events were observed in the groups with higher serum zinc concentrations (G3 and G4 groups) than in the groups with lower serum zinc concentrations (G1 and G2 groups). No events were observed during the three-year observation period in patients with higher platelet counts and higher serum zinc concentrations. The same trend was observed in the HCC incidence rate (Figure 6c,d). In summary, lower cumulative incidence rates of events and incidence rates of HCC were observed in the Zn-group than in the no Zn-treatment group, and this effect was much more clearly observed in patients with serum zinc concentrations exceeding 70 µg/dL after Zn supplementation.

## 4. Discussion

Serum zinc concentration gradually decreases as liver fibrosis progresses, and zinc supplementation suppressed the exacerbation of the disease in patients with CLDs. According to these results, a reduction in zinc levels partially causes the worsening of liver function. In other words, many liver enzymes do not work well because of a zinc deficiency. The importance of zinc in the urea cycle has been elucidated; many investigators have shown that zinc supplementation improves hepatic encephalopathy after short-term treatment (7 days–3 months). Zinc is needed for the activation of not only OTC in the urea cycle, but also many other enzymes, such as DNA polymerase, RNA polymerase, alkaline phosphatase and SOD; thus, researchers have speculated that the administration of zinc may exert a positive effect on liver function and inhibit cancer development. It is difficult to evaluate the influence of zinc supplementation on liver function and the incidence rate of HCC in the short term; therefore, the period of zinc administration and the follow-up period were longer than 3 years in this study, enabling us to address this issue. In the present study, we revealed that supplementation with zinc was effective at maintaining liver function and suppressing HCC. These results are quite interesting. The mechanism by which zinc suppresses the deterioration of liver function and the development of HCC is currently unknown, but three possibilities exist. First, improvement of liver injury is caused by normalization of both extracellular ATP clearance and adenosine generation [1]. Second, SOD activity is restored to the basal level, enabling reductions in the levels of ROS, which can induce epigenetic modulations that lead to carcinogenesis. In support of this hypothesis, we examined the value of dROM, which is a possible marker of ROS [13,14]. In almost all the zinc-treated patients, the value of dROM was significantly decreased after the administration of zinc (unpublished data). Third, the severity of fibrosis is related to the risk of HCC development, and Takahashi reported that the serum levels of type IV collagen and the activity of tissue inhibitors of metalloproteinase-1 (TIMP-1) were significantly reduced by oral zinc supplement therapy [15]. Zinc therapy can suppress the progression of fibrosis, which reduces the risk of HCC development.

It is necessary to determine the serum zinc concentration that is needed after zinc administration to obtain a better prognosis. In this study, we revealed that a serum zinc concentration of 70 µg/dL after zinc supplementation was needed to exert this inhibitory effect on HCC development. Few papers have explored the appropriate serum zinc concentration. Takamatsu reported that serum zinc concentrations greater than 72 µg/dL are needed to suppress fibrosis, based on a study with 57 patients with CLDs [16]. Our results are consistent with this report, and both studies showed that it is important to increase and maintain a certain serum zinc concentration to obtain a better prognosis.

This is the first report to clarify that zinc administration improves liver function and decreases the cumulative incidence of events and the incidence of HCC in patients with CLDs, over the course of long-term follow-up. We also confirmed that a serum zinc concentration greater than 70 µg/dL must be maintained to obtain good clinical outcomes, and patients should take more than 90 mg/day of zinc to achieve the aforementioned serum zinc concentration. The effectiveness does not depend on the etiology of the CLD; thus, zinc administration is equally useful for patients with an HCV infection, HBV infection, NASH, or alcohol-related liver diseases. It is necessary to highlight the importance of zinc and to start administering zinc preparations in the clinical setting.

## Figures and Tables

**Table 1:** Clinical profiles of 267 patients with chronic liver diseases enrolled in this study. F/M: Female/Male; ns: not significant; HCV/HBV/alcohol/NASH/AIH+PBC: hepatitis C virus/hepatitis B virus/alcohol/nonalcoholic steatohepatitis/autoimmune hepatitis + primary biliary cholangitis.

	Zn Group	No Treatment Group	*p*-Value
number of patients	196	71	
mean age (years)	73.2 ± 9.5	66.4 ± 12.6	*p* < 0.0001
gender (F/M)	97/99	36/35	ns
chronic hepatitis/liver cirrhosis	93/103	56/15	*p* < 0.0001
Child-Pugh (A/B/C)	98/89/9	67/4/0	*p* < 0.0001
Zn (μg/dL)	51.0 ± 16.8	61.7 ± 9.1	*p* < 0.0001
T-Bil (mg/dL)	1.2 ± 1.8	0.8 ± 0.4	*p* < 0.0001
albumin (g/dL)	3.5 ± 0.6	4.0 ± 0.3	*p* < 0.0001
PT activity (%)	77.8 ± 17.6	89.1 ± 11.7	*p* < 0.0001
platlet count (10^4^/L)	12.9 ± 14.2	15.8 ± 7.1	*p* = 0.035
observation period (Months)	40.0 ± 31.5	39.6 ± 22.8	ns
Etiology (HCV/HBV/alcohol/NASH/AIH+PBC)	121/10/23/19/23	35/13/7/7/8	ns

**Table 2:** Clinical characteristics of the 4 groups stratified by their serum zinc concentrations at 6 months after the start of the zinc treatment.

	G1 Group <50 μg/dL	G2 Group ≥50 and <69 μg/dL	G3 Group ≥70 and <89 μg/dL	G4 Group ≥90 μg/dL	*p*-Value
number of patients	23	42	38	93	ns
mean age (years)	75.0 ± 7.3	72.6 ± 8.9	74.1 ± 10.2	72.6 ± 10.0	ns
gender (F/M)	14/9	21/21	21/17	43/50	ns
chronic hepatitis/liver cirrhosis	6/17	20/22	18/20	47/46	ns
Child-Pugh (A/B/C)	8/14/1	21/20/1	17/20/1	52/35/6	ns
Zn (μg/dL) (before treatment)	49.8 ± 18.0	51.3 ± 11.5	49.4 ± 13.3	52.0 ± 20.1	ns
T-Bil (mg/dL)	2.2 ± 2.8	1.5 ± 0.9	1.5 ± 0.9	1.5 ± 2.2	ns
albumin (g/dL)	3.1 ± 0.4	3.3 ± 0.5	3.1 ± 0.6	3.3 ± 0.6	ns
PT activity (%)	66.2 ± 16.2	68.2 ± 19.9	69.0 ± 15.0	72.0 ± 17.7	ns
platelet count (10^4^/L)	10.4 ± 11.7	11.5 ± 6.9	13.3 ± 14.6	14.2 ± 16.9	ns

## Supplementary Materials

The following are available online at http://www.mdpi.com/2072-6643/10/12/1955/s1, Figure S1: Serial changes of AST and ALT values in the Zn- and no Zn-treatment groups; Table S1: Clinical backgrounds in the Zn- and no Zn-treatment groups.

## Abbreviations

CLDs, chronic liver diseases; HCC, hepatocellular carcinoma; OTC, ornithine transcarbamylase; GDH, glutamate dehydrogenase; SOD, superoxide dismutase; T-Bil, total bilirubin; Alb, albumin; PT, prothrombin activity; BTR, branched chain amino acids/tyrosine ratio; TIMP-1, tissue inhibitors of metalloproteinase-1.
