# Supplementary material for: Long-Term Zinc Supplementation Improves Liver Function and Decreases the Risk of Developing Hepatocellular Carcinoma
Source: Nutrients. 2018 Dec 10;10(12):1955. doi: 10.3390/nu10121955 (PMC6316561; doi:10.3390/nu10121955)
Supplement: Supplementary file 1 [file nutrients-10-01955-s001.zip › nutrients-383228 Supplementary Table S1.pdf]

Supplementary Table 1

		Zn group	No treatment group	p-value
the number of patients		196	71	
alcohol consumption (no/small/large)		164 / 6 / 26	60 / 3 / 8	ns
DM (with/without)		53 / 143	12 / 59	p=0.054
BMI (kg/m^2)		22.4±4.1	22.2±3.6	ns
Attending physician (Dr. A/Dr. B/Dr. C/others)		72 / 66 / 11 / 47	32 / 28 / 5 / 6	ns
concomitant drugs	BCAA (with/without)	150 / 46	51 / 20	ns
	Diuretics (with/without)	82 / 114	3 / 68	p<0.0001
	UDCA (with/without)	123 / 73	44 / 27	ns

Alcohol consumption, small: 0< <60g/day, large: 60g ≦/day
BCAA: branched-chain amino acid
UDCA: ursodeoxycholic acid
