# Supplementary figures and images for: Long-Term Zinc Supplementation Improves Liver Function and Decreases the Risk of Developing Hepatocellular Carcinoma
Source: Nutrients. 2018 Dec 10;10(12):1955. doi: 10.3390/nu10121955 (PMC6316561; doi:10.3390/nu10121955)

# Supplementary Figure 1

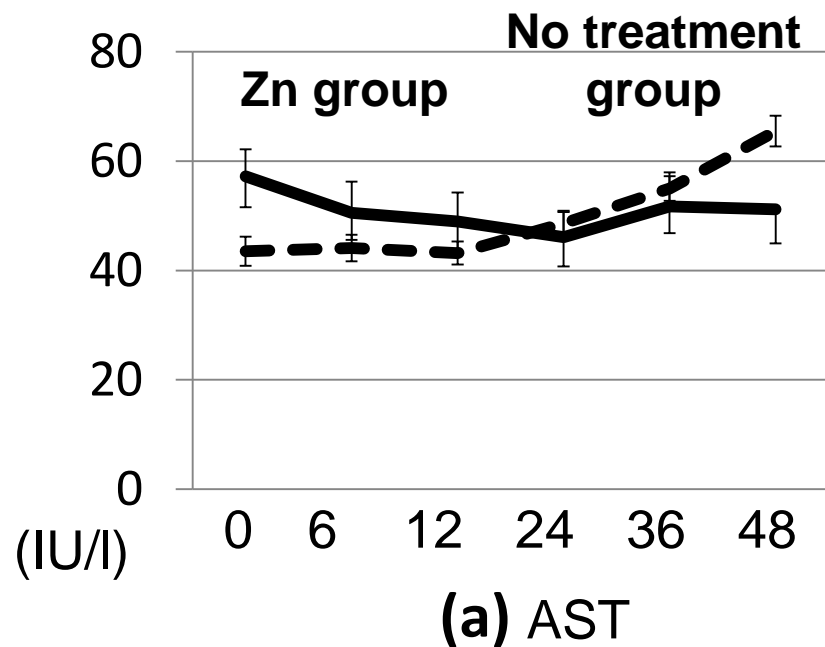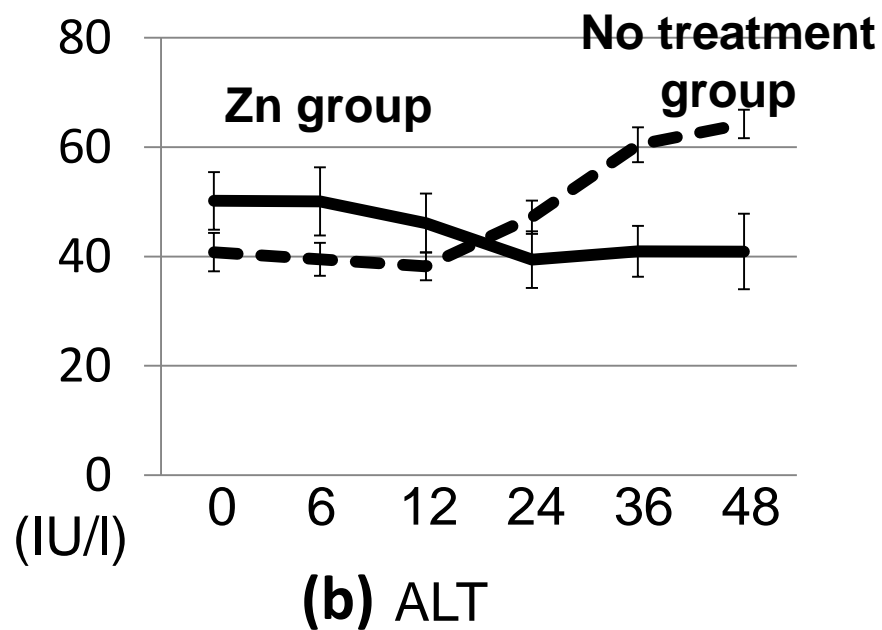

Supplement: Supplementary file 1 [file nutrients-10-01955-s001.zip › nutrients-383228 Supplementary Figure S1.pdf]
